# Executive Function and Sensory Processing in Dichotic Listening of Young Adults with Listening Difficulties

**DOI:** 10.3390/jcm10184255

**Published:** 2021-09-19

**Authors:** Aline Tocchini Pascoinelli, Eliane Schochat, Cristina Ferraz Borges Murphy

**Affiliations:** Department of Physical Therapy, Speech-Language Pathology and Occupational Therapy, School of Medicine, University of São Paulo, São Paulo 05360-160, Brazil; aline.tocchini@gmail.com (A.T.P.); eschocha@usp.br (E.S.)

**Keywords:** auditory processing disorder, working memory, inhibitory control, dichotic tests

## Abstract

Previous studies have suggested that varying attention demands in dichotic listening (DL) tasks might be a clinically feasible method to distinguish ‘bottom-up’ from ‘top-down’ deficits in listening. This study aims to investigate DL processing in adults with listening difficulties (LD). We assessed the performance of a listening difficulties group (LDG) (*n* = 24, mean age = 24, backward digit span = 4.0) and a control group (CG) (*n* = 25, mean age = 29.2, backward digit span = 6.4) in DL tests involving non-forced and both right and left-forced attention. The results indicated an overall significantly worse performance of LDG compared to the CG, which was greater for forced-left condition. This same result was observed when controlling for working memory (WM) variance. Both groups presented an overall right ear advantage with no difference in terms of the magnitude of advantage. These results indicate that LD presented by the LDG might be due to a combination of sensory and cognitive deficits, with emphasis on the cognitive component. However, the WM, although impaired in the LDG group, was not the main factor in segregating both groups. The role of the additional cognitive processes such as inhibitory control in LD is discussed.

## 1. Introduction

Despite decades of research, auditory processing disorder (APD) is still a topic of intense debate and controversy [1,2,3,4,5]. The controversy has mainly involved the lack of strong evidence of impaired processing in the auditory system [4], the heterogeneous nature of APD, with varied symptoms and extensive comorbidities [6,7,8], and the lack of a gold standard for diagnostic accuracy [9], including the challenge involving the influence of high-order cognitive functions on auditory processing tasks [8,10].

One promising approach that might bring new evidence of top-down control processes on the auditory system is the dichotic listening paradigm (DL), more specifically, the clinical use of non-forced and forced recall on DL tests. The non-forced recall involves reporting sounds heard in either ear or both, whereas forced recall asks for sounds heard in a specific ear to be reported. It is well established that in the non-forced recall, the most common condition used clinically [11,12,13,14,15], right-handed individuals generally report stimuli from the right ear more accurately, compared to the left ear, leading to a right ear advantage (REA). This perceptual saliency was initially explained by the “structural model” [16]. According to this model, an REA occurs due to the left hemisphere dominance for speech processing together with a stronger contralateral than the ipsilateral neural projection from the ears to the cerebral cortex. This has been most commonly studied using syllable stimuli (e.g., /da/, /ba/). Research has shown that, using forced recall, a top-down attention modulation of the REA effect is elicited [17]. More specifically, when the individual has to focus attention on the right ear stimulus, the REA significantly increases as the bottom-up “perceptual saliency” of the stronger, crossed ascending auditory pathway is boosted by top-down attention focus on the same right ear. On the other hand, when attention is focused on the left ear, there is a conflict between perceptual saliency of the right ear and top-down attention focus on the left ear. This “cognitive control model” was proposed and has been extensively studied by Hudghal and colleagues [18]. Imaging studies [19,20] addressing neural correlates of ear advantage on DL tests have supported the idea that the “cognitive control model” would complement the “structural model,” demonstrated by the interaction between sensory and attentional activation components in mainly frontal and parietal cortical areas. These imaging studies were supported by attentional and sound level modulation of DL ear advantage.

A considerable number of studies have investigated the performance on DL tests of clinical groups with primary top-down impairments in both non-forced and forced-attention conditions. The clinical groups have included mostly individuals with executive impairment, such as schizophrenia [21,22,23] and ADHD [24], or language disorders, such as stuttering [25,26,27] and reading difficulties [28]. Dramsdahl et al. [24], for example, investigated an ADHD group’s performance on non-forced and forced-attention conditions and demonstrated that, compared to a control group, the ADHD group had a significantly poorer performance only on forced-left attention. This result was interpreted as owing to the cognitive conflict triggered by the forced-left condition and the executive function impairment presented by children with ADHD. Blood et al. [25], comparing a stuttering and control group’s performance on DL tests, demonstrated less accurate responses of the stuttering group when they were asked to report the digits presented to the left ear, suggesting attentional direction difficulties.

Few studies have investigated the performance of groups with listening difficulties in dichotic listening tests using different attention conditions [29,30,31]. Jerger and Martin [30] applied forced and non-forced conditions in a group of elderly individuals presenting some degree of age-related hearing loss (mild to moderate), a presumably bottom-up dominated problem. The results indicated different patterns of performance, namely a small subgroup (19%) showing normal results for both non-forced and forced conditions, a second subgroup (23%) presenting abnormal results for both conditions (forced-left and non-forced condition, mainly in the left), and the largest and last subgroup (58%) showing abnormal results only in the left ear for non-forced attention. The authors considered each pattern to hypothesize a different degree of cognitive control on listening skills. The second subgroup’s performance, for example, was interpreted based on the cognitive control model. The authors suggested that the left-ear deficit for both conditions might result from an inability to recruit or allocate appropriate executive functions. Following this rationale, they also highlighted the potential of applying those different conditions clinically, as they might bring valuable insights regarding the contribution of cognitive and auditory-specific factors to listening.

The studies reviewed here have demonstrated favourable results regarding the potential of both forced and non-forced conditions as a psychophysiological model to distinguish “bottom-up” (via non-forced condition) from “top-down” (via forced-left condition) deficits. Conversely, one of the main challenges involving auditory processing disorder is dissociating sensory from cognitive components of listening to characterize their specific role in this disorder. This dissociation is fundamental given the two main hypotheses concerning the nature of listening difficulties: disordered processing involving the central auditory system (bottom-up nature) or a primary executive function/attention impairment (top-down nature). To our knowledge, only one recent study [29] used this approach in children with listening difficulties. Moore et al. [29] compared children’s performance with and without listening difficulties in non-forced and forced DL tests using varied levels of interaural difference between stimuli. While no difference was observed between groups in all conditions with no interaural level difference (ILD), there was significantly higher laterality in the group of listening difficulties for conditions with large ILD favouring the left ear. The authors discussed different hypotheses to explain those results, such as the presence of a primary auditory problem or a deficit to balance greater sound levels through attention modulation in the group of children with listening difficulties. 

The current study also aimed to compare the performance of individuals with listening difficulties (LDG) and a control group in non-forced and forced DL tests. However, we included adults instead of children to rule out maturational effects. In addition, different parameters were included to examine the influence of multi-faceted linguistic and cognitive demand variation for each condition. Thus, tasks with syllables × digits as well as two pairs × three pairs of stimuli were applied in each one of those three different conditions (non-forced, forced-right and forced-left). A backward digit span test was also included to index working memory and executive function for each group and facilitate interpretation of the relation between cognitive function and DL performance. 

Based on the literature described, we predicted that the LDG would present less accurate responses, in general, compared to CG. More specifically, difficulty in the non-forced conditions would point to a primary auditory problem, while difficulty in forced conditions (especially the forced-left) would indicate that the listening difficulties’ primary cause also has a top-down nature. The influence of those different parameters will be discussed for each condition.

## 2. Materials and Methods

This study was conducted at the Department of Physical Therapy, Speech-Language Pathology and Occupational Therapy in the School of Medicine at the University of São Paulo and was approved by the Research Ethics Committee of the Analysis of Research Projects of the Hospital das Clínicas, Medical School, the University of São Paulo under protocol number 116/11. A written consent form with detailed information about the aim and the protocols of the study was also approved by this ethics committee. All participants provided written informed consent before participation in the study.

### 2.1. Participants

A total of 49 individuals, comprising 2 different groups, participated in the study: the listening difficulties group (LDG), composed of adults with primary listening complaints (*n* = 24, mean age = 24 years old), and a control group (CG) (*n* = 25, mean age = 29.2 years old).

The inclusion and exclusion criteria were investigated by applying a general questionnaire including sociodemographic/medical and educational questions. The inclusion criteria for both groups were: age between 18 and 55 years, either gender, right-handed, no history of neurological disorders or injuries, psychiatric, cognitive, or developmental disorders. The CG was also required to have no history of otology/listening problems, such as recurring otitis media with effusion and previous/current concerns regarding understanding speech in silence and background noise. After investigating those criteria, an audiological assessment was carried out to confirm the normal hearing sensitivity of all participants. The inclusion criteria were otoscopy indicating no abnormalities, Type A at the Tympanometry, and pure-tone thresholds ≤20 dB HL from 500 Hz to 8 kHz, without asymmetrical hearing levels, at pure-tone audiometry (PTA),The PTA was conducted in a Siemens soundproof booth, using a GSI-61 two-channel clinical audiometer. The stimuli were presented via headphones TDH39.

The listening difficulties group (LDG; *n* = 24, mean age = 24 years old) was composed of participants referred to the audiology clinic due to their primary concern involving listening skills. After confirming normal hearing sensitivity through the audiological assessment, comprehensive auditory processing (AP) questionnaire was carried out to assess the listening difficulties in detail (Table 1). This decision to use this AP questionnaire, instead of auditory processing tasks, was based on the challenge of dissociating sensory from cognitive components of listening through auditory processing tasks [8,9,10]. Conversely, the questionnaire could indicate whether those symptoms reported would involve auditory sensory skills predominantly or top-down symptoms in nature. The questionnaire was developed specifically for this research as no other similar tool is available in Portuguese and it was comprised of questions involving specific auditory sensory (Section 1) and top-down skills involving memory, attention, and language (Section 2). The selection of those questions was based on a questionnaire already published and clinically validated for the adult population [32,33]. The participant was instructed to give an affirmative or negative answer for the presence of the symptoms in each session. One point was given for each answer “yes”. The total score in each session indicated the severity of bottom-up and top-down difficulties. Thus, the “predominant listening difficulties” criterion was defined as the combination of a high score in Session 1 + a low score in Section 2. This was represented by the cut-off scores Section 1 ≥ 5 points and Section 2 ≤ 3 points. This criterion would indicate, quantitatively, individuals with predominantly auditory sensory symptoms. The result shown in Table 1 present the most and the least common symptoms reported in both sections. 

A backward digit span test was used to index the working memory and executive function of all participants. Participant characteristics are shown in Table 2. There was no significant difference between the mean age of both groups. Regarding the backward digit span test, there was a significant difference between their performance (*p* < 0.01), with CG presenting the better performance.

### 2.2. Dichotic Listening (DL) Tests

A series of DL tests were carried out in a soundproof booth. The stimuli were delivered via a compact disc player, connected to an audiometer, and headphones. The stimuli were presented at 70 dB HL. The characteristics of the tests were as follows:

#### 2.2.1. Dichotic Digit Test with 2 Pairs of Digits 

This test was developed in 1996 [34,35], and it is based on the American dichotic digit test proposed by Musiek [11]. Clinically, it is widely used in Brazil as it contains normative data for children and adults. The task consists of 12 trials, each composed of 2 successively presented pairs of digits, randomly selected from the five disyllabic digits of Brazilian Portuguese (4,5,7,8,9). Only those five digits were selected because they are all disyllabic words and presented a similar duration. Despite that, when performing the test, the tester did not inform the listener which digits were included, enabling a wide range of possibilities as an answer. The stimuli were recorded in a professional studio by a professional speaker and were scaled for audibility. Each digit pair was simultaneously presented in the right and left ear, and the two digits within a pair were always different, characterizing a dichotic task.

#### 2.2.2. Dichotic Digit Test with 3 Pairs of Digits

This test was an adaptation of the test described above (Dichotic Digit Test with 2 pairs). The third pair of randomly chosen digits was added to each trial to increase memory load. Digits were prepared and scheduled using the audio editor software Sound Forge 9.0. The task also consisted of 12 trials (3 pairs of digits each).

#### 2.2.3. Dichotic Syllable Test with 2 Pairs of Syllables

This test was developed in 1995 [36,37], and it was based on the test proposed by Pinto in 1991. The task also included 12 trials, each one composed of 2 pairs of syllables. The stimuli were six syllables of Brazilian Portuguese, each one composed of plosive consonants and the vowel “a” (/ga/, /ta/, /ca/, /da/, /ba/, /pa/). The vowel “a” was selected because, according to spectrographic analysis, it is the most stable vowel in the Brazilian Portuguese language [38]. Plosives, on the other hand, have been shown to elicit a significant right ear advantage [39]. All combinations between syllables were included except for pairs with the same syllables (eg., /ga/ /ga/) and same sonority (eg., /pa/ /ba/, /ta/ /da/, /ka/ /ga/). A second task with 3 pair of syllables was also applied; details as per digits.

#### 2.2.4. Dichotic Syllable with 3 Pairs of Syllables in 12 Trials

This test extended the Dichotic Syllable Test by adding a third pair of syllables.

**Figure 1:**
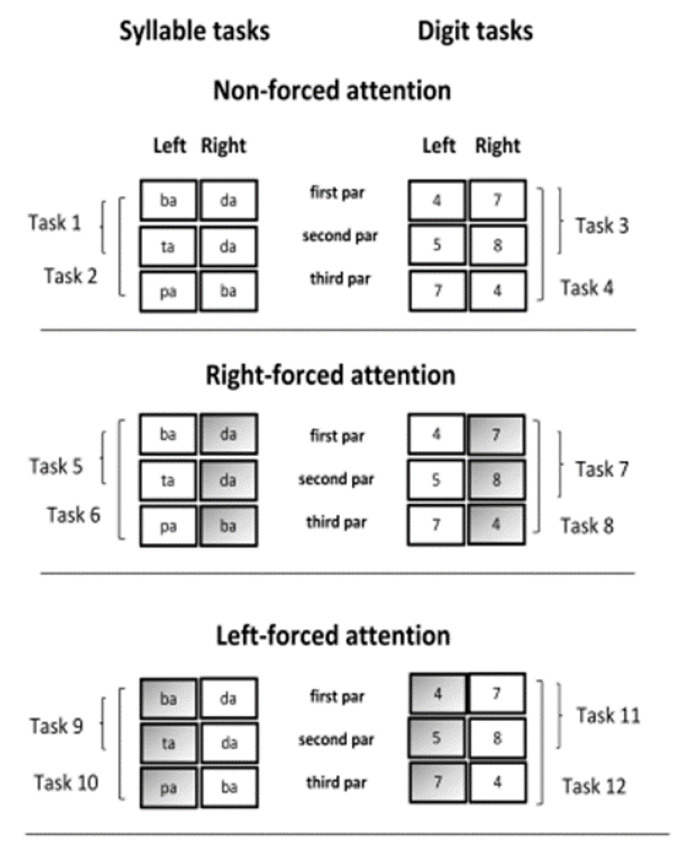
Characteristics of the 12 DL tasks applied.

Each of these four dichotic tests was applied under non-forced, forced-right, and forced-left conditions, totaling 12 different tasks (Figure 1).

The tests were carried out in a random order to avoid training-related biases. In the non-forced condition, participants were instructed to report only one digit from each pair presented, the one they heard best and most clearly. This instruction was based on previous studies involving dichotic listening [40,41]. However, in those studies, only one pair of stimuli was presented per trial. In the current study, for tasks with 2 pairs of stimuli per trial, 2 stimuli should be repeated per trial and for tasks with 3 pairs of stimuli, 3 stimuli should be repeated per trial. Participants were instructed to focus on digits presented to the right or left ear in the forced-right and forced-left conditions, respectively, and report one digit or syllable from each pair presented per trial. The variable was the number of correctly reported digits or syllables for each ear.

### 2.3. Statistical Analyses

Firstly, we compared the groups for the variables “mean age,” and “backward digit span performance”, performing one-way ANOVAs. Then, a four-way mixed ANOVA with repeated measures was performed with group (CG, LDG) as between-measure and conditions (non-forced, forced-right, forced-left), type of stimuli (syllables × digits) and number of stimuli (2 × 3 pairs) as within-measures. This same analysis was also performed regressing out the variable backward digit span (ANCOVA) to facilitate discussion regarding the influence of working memory on those tasks. For the non-forced condition, an “ear effect” analysis was also performed, including an additional analysis for the Laterality Index × group interaction. Laterality index (LI) is defined as the number of correctly reported left or right ear stimuli, expressed as a percentage as LI = 100 (R − L)/(R + L). A positive LI is an REA. The significance level was set at *p* < 0.05.

## 3. Results

**Figure 2:**
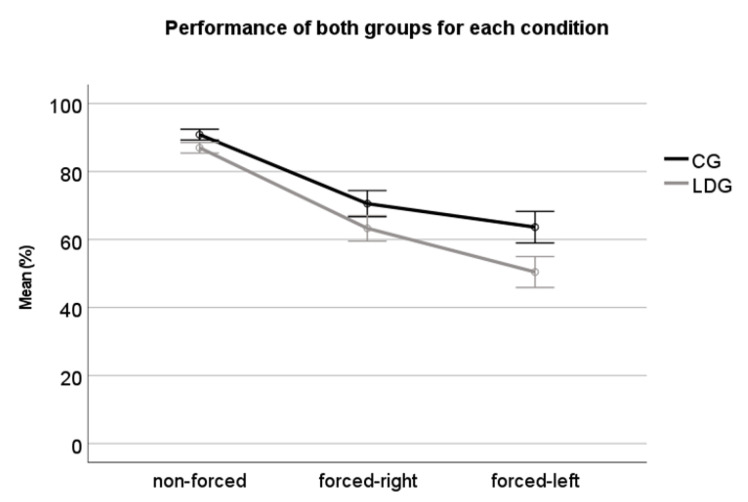
Group’s performance for non-forced condition (NF), forced-right condition (FR) and forced-left condition (FL). CG: control group; LDG: listening difficulties group.

The results indicated an overall “group effect” (F(1,43) = 18.93, *p* < 0.01, ηp2 = 0.306), with the LDG presenting less accurate responses than the CG. There was also a “condition effect” (F(1,43) = 464.4 *p* < 0.01, ηp2 = 0.91), with post-hoc analysis (Bonferroni) indicating a significant better performance for non-forced condition (*p* < 0.01), followed by forced-right (*p* < 0.01) and forced-left (*p* < 0.01). An interaction between group x condition (F(1,43) = 9.93, *p* < 0.01 ηp2 = 0.18) was also detected, which is shown in Figure 2.

This interaction was further teased out through additional ANOVA. The results indicated a significant difference between both groups’ performance for all conditions (non-forced: (F(1,43) = 12.15, *p* < 0.01); forced-right: (F(1,43) = 7.38, *p* < 0.01); forced-left: (F(1,43) = 16.72, *p* < 0.01). Thus, those results indicate that, although the LDG has had the worst performance at all conditions, the difference between the groups becomes progressively larger for forced-right and left conditions.

To investigate the extent to which cognitive function (working memory) and sensory components of DL affected the results, an additional analysis (ANCOVA) was performed regressing out the variance associated with the backward digit span task. The results were very similar to the previous ones: there was still a group effect (F(1,42) = 8.157, *p* < 0.01), with the LDG presenting less accurate responses than the CG; a condition effect (F(1,42) = 24.36, *p* < 0.01), with Post-hoc analysis (Bonferroni) indicating a significant better performance for non-forced condition (*p* < 0.01), followed by forced-right (*p* < 0.01) and forced-left (*p* < 0.01) and an interaction between group x condition (F(1,42) = 5.89, *p* = 0.02), with a similar pattern of performance between groups.

**Figure 3:**
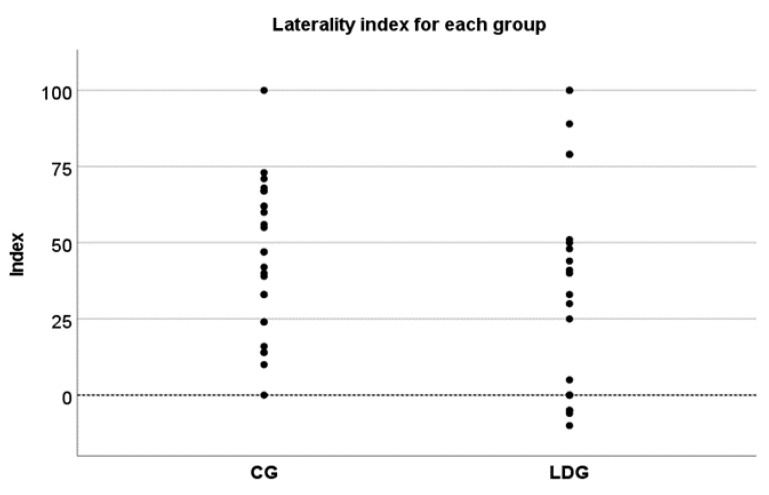
Degree of lateralization of each group with positive index representing right ear advantage. The groups showed similar magnitude of right ear advantage, with few participants of LDG demonstrating a left ear advantage (negative index).

An overall ear effect was also observed (F(1,43) = 52.4, *p* < 0.01, ηp2 = 0.550), with greater scores for the right ear. This effect was observed regardless of the group (F(1,43) = 52.4, *p* < 0.01, ηp2 = 0.550), which indicates a similar “right ear advantage” (REA) for all participants. The degree of magnitude of the right ear advantage was also investigated for each group through the laterality index. ANOVA revealed no group effect (F(1.43) = 0.692, *p* = 0.41), demonstrating an overall similar magnitude of REA regardless of group. Despite that, four participants in the LDG presented left ear advantage, which was indicated by the negative number (Figure 3).

**Figure 4:**
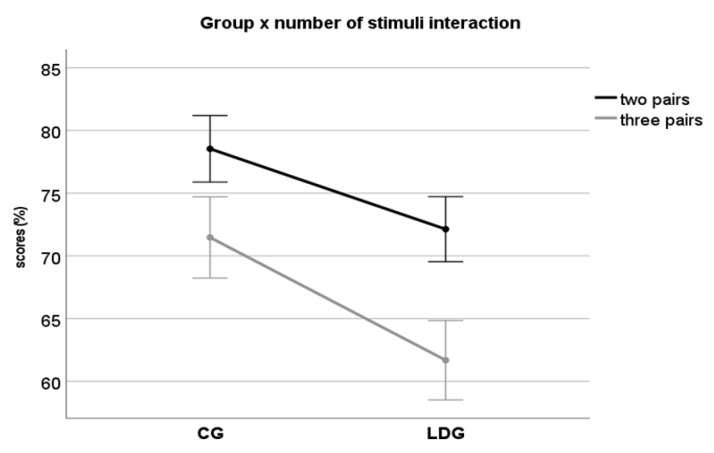
Interaction between group × “number of stimuli” variable. Although the LDG has had worse performance than CG for tests with 2 and 3 pairs, the difference between group was larger for 3 pairs.

The “type of stimuli” and “number of stimuli” effect was also investigated across groups and conditions. The result demonstrated a general “type of stimuli” effect (F(1,43) = 938, *p* < 0.01, ηp2 = 0.95), with better performance for digits compared to the syllables regardless of the group (F(1,43) = 0.361, *p* = 0.55), which means that this pattern was similar for both groups. A general “number of stimuli” effect was also observed (F(1,43) = 102, *p* < 0.01, ηp2 = 0.70), with better performance for tasks with two pairs compared to 3 pairs of stimuli. An interaction between group × number of stimuli was observed (F(1,43) = 3.80, *p* = 0.05, ηp2 = 0.08), with a larger difference between groups for tests with three pairs, in which the LDG presenting worse performance (Figure 4).

The same analysis involving type and number of stimuli was performed regressing out the variance associated with the backward digit span task. The results indicated similar “type of stimuli” effect (F(1,43) = 35.1, *p* < 0.01) and “number of stimuli” effect (F(1,43) = 4.34, *p* = 0.04). However, no interaction was observed between group × number of stimuli (F(1,43) = 2.71, *p* = 0.10).

## 4. Discussion

This research aimed to examine executive and sensory processing in individuals with listening difficulties through a series of DL tests. The results demonstrated an overall significantly worse performance of LDG, which was more evident for forced conditions, especially the forced-left. The same result was observed when controlling for backward digit span variance. Both groups also presented overall similar right ear advantage. However, a small subgroup of LDG presented left ear advantage. A similar pattern of results between groups was observed for both digit and syllables. Regarding the number of stimuli, the LDG presented a significantly worse performance for tests with three pairs. However, this difference disappeared when controlling for backward digit span variance.

Firstly, the backward digit span test’s performance demonstrated that executive function, more specifically, working memory, is impaired in the LDG. This finding supports previous research that revealed a link between executive function and listening difficulties [7,8,10,42,43]. Several studies have demonstrated not only the poor performance of children with listening difficulties in cognitive tests (including working memory tests), but also the cognitive performance as the best predictor of poor listening [7,8,10,42]. The responses obtained through the current questionnaire also indicate that, even when the main concern involves listening skills, memory and attention issues might also be present. The only study which investigated cognitive skills in adults with listening difficulties also indicated a low score in an auditory memory test [43], corroborating the current findings.

The poor performance of LDG, compared to the CG, at all conditions might indicate that this group might have both sensory and cognitive impairments, as demonstrated by the combination of symptoms presented in the questionnaire. However, it would also show that cognitive control processes might be more significantly impaired, given the greater difference between both groups for forced conditions, especially forced left. The larger difference for forced left condition might be explained by different degrees of cognitive conflict elicited by each specific forced task. Studies involving neural correlates have found that different neural mechanisms may underpin each forced condition [19,20,44]. Thomsen et al. [44] showed activation of the superior temporal gyrus, middle and inferior frontal gyrus, and the cingulate cortex in both forced conditions. However, there was an increase of activation for forced left. Those findings could explain why this specific condition was more sensitive to distinguish between both groups. 

To further investigate the underlying factors involved in each task, we regressed out working memory variance. The results still indicated a significant difference between groups for all conditions. This suggests that this specific aspect of executive function was not the main factor in segregating both groups, even for forced conditions. One hypothesis is that forced conditions might encompass additional executive cognitive processes that might be involved with listening difficulties and were not controlled in the present study. Forced-left condition, for instance, involves the conflict between the perceptual saliency effect demonstrated by the REA and the instruction to focus attention on the opposite ear (left ear). This is not only related to focused attention, as observed in the forced-right condition, but also inhibitory control in an ambiguous situation [45]. Thus, it is possible that the inhibitory control process could have effectively differentiated LDG from CG. This hypothesis is more plausible when one specific aspect of inhibitor control is taken into account, the inhibitory control of attention. This process is related to selectively attending to one stimulus while suppressing attention to the other one [46]. This selective attention skill is required, for instance, when the individual has to focus on one auditory message and ignores others presented simultaneously as a background message. This is a common situation which people with listening difficulties refer to as a struggle: focusing on one auditory message while ignoring others presented simultaneously. In fact, this was the most common symptom described by the LDG in the questionnaire applied in the current study. Bamiou et al. [47] also reported that non-neurological adults with listening difficulties scored worse in one specific question from an APD questionnaire, related to how well they could ignore sound when listening to something else. Based on this finding, they discussed that the instruction to focus attention on one ear in dichotic digit tests should be clinically more appropriated for this population, which is in line with the current results on the forced-left condition. Further studies should investigate inhibitory control of attention in individuals with listening difficulties to clarity its role in listening difficulties.

The results also indicated a similar right ear advantage (REA) for both groups and a small LDG subgroup with left ear advantage. Research involving EA in listening difficulties group also showed conflicting results, such as a left-ear advantage [5,20] or a significantly larger REA [48]. Basically, the American Academy of Audiology [13] stated that an exaggerated REA or LEA has implications on APD in children. However, it is also reported that children usually have naturally larger ear advantages [49], possibly as a reflex of the immaturity of both sensory and attentional systems [20]. Thus, it is likely that the sensitivity of the dichotic tests is higher for groups of children in general rather than adults. This might be the reason why the overall laterality index was similar in both groups. The presence of the small group with LEA might indicate differences in terms of the degree of sensory and supramodal deficits between those individuals with listening difficulties, as indicated by Schmithorst et al. [20]. Another relevant point is that around 15–20% of right-handed individuals might exhibit no ear or left ear advantage, as demonstrated in the current research [50]. Thus, further studies with larger samples should investigate this aspect to examine whether it is a characteristic of the listening difficulty.

Another current finding was related to the effect of type and number of stimuli. Regarding the type of stimuli, the groups, in general, exhibited the same pattern of responses: better performance for digits than syllables. Different performances for different types of stimuli are expected as each stimulus reflects different stages of speech processing [51]. The greater performance for digits probably reflects the positive interference of linguistic cues (semantic properties), making the stimuli more redundant, and consequently easier to understand than syllables. Musiek and Chermak [52] have explained that digits are more familiar and highly intelligible than CV. Thus, as consequence, this familiarity and intelligibility effect differed both stimuli in terms of level of difficulty to be perceived. The presence of the same pattern of performance between groups indicates that this linguistic factor seems not to be involved in the nature of the listening difficulties presented by the LDG. On the other hand, the number of stimuli effect was different for each group. Both groups presented a better performance for two rather than three pairs of stimuli. However, the LDG presented significantly lower scores for three pairs of digits, an interaction that disappeared when controlling for working memory variance. This confirms the negative effect of working memory deficit on tests involving several stimuli. This result is clinically important as it shows how supramodal deficits might directly impact the results of auditory processing tests. 

This study has some limitations. The LDG was selected through a new, non-validated questionnaire. Thus, further studies should use validated questionnaires with norm data and cut-off scores to confirm the current results. In addition, the sample size of each group was small, which might have affected the finding of more evident results. Thus, the current results should also be confirmed with larger sample sizes.

## 5. Conclusions

The overall poor performance of the LDG at all DL conditions suggests the listening difficulties presented by the LDG might be due to a combination of sensory and cognitive deficits, although the more significant difference in the forced-left condition has highlighted the cognitive factor. Although impaired in the LDG group, the working memory was not the main factor segregating both groups. The role of additional cognitive processes such as inhibitory control in listening difficulties should be investigated in further studies.

## Figures and Tables

**Table 1:** Questionnaire about listening, memory, attention and language skills.

	Number of Subjects from LDG Answering “Yes” or “No”	
	Yes	No
Section 1—Questions about listening skills		
1. Do you struggle to understand people when there is background noise?	23	1
2. Do you feel that you have to make an effort to follow a long conversation?	14	10
3. Do you struggle to localize sounds? e.g., to know which direction a siren sound is coming from.	6	18
4. Do you usually ask people to repeat what they told? e.g., you frequently say what? Huh? Pardon?	19	5
5. Do you feel that you need extra time to process what was told? e.g., you take time to understand a verbal explanation or instruction	12	12
6. Do you feel much easier to understand written rather than verbal information? e.g. to read a text rather than attending a lecture about the same topic	8	16
7. Do you struggle to ignore one auditory message when you try to listen to another one at the same time?	22	2
Section 2—Questions about memory, attention and language skills		
1. Do you have memory difficulties in general? e.g., to remember where you left something, to forget what you should buy in the shop, forget the tasks you have to do, appointments or important dates.	9	15
2. Do you struggle to do more than one task at the same time?	8	16
3. In general, do you struggle to focus? e.g., struggle to keep focused when you watch a movie, read books.	16	8
4. Do you easily get distracted and are constantly daydreaming?	13	11
5. Do you struggle with organization? e.g., your room, work desk, or car is always messy.	13	11
6. Do you struggle with reading comprehension? e.g., you have to reread a text several times to understand the meaning	8	16
7. Do you usually mix up letter or numbers when reading or writing?	2	22

**Table 2:** Characteristics of the participants.

Variables	CG (*n* = 22)	LDG (*n* = 23)
Age (M ± SD)	27 ± 5.9	24.3 ± 4.2
Executive task		
Backward Digit Span	6.4 ± 1.5	4.0 ± 1.0
Auditory task		
Audiological evaluation	Normal	Normal
